# Case Report: Superior Vena Cava Resection and Reconstruction for Invasive Thyroid Cancer: Report of Three Cases and Literature Review

**DOI:** 10.3389/fsurg.2021.644605

**Published:** 2021-06-01

**Authors:** Wenjie Chen, Jianyong Lei, Yichao Wang, Xiaojun Tang, Bin Liu, Zhihui Li, Qinghua Zhou

**Affiliations:** ^1^Thyroid and Parathyroid Surgery Center, West China Hospital of Sichuan University, Chengdu, China; ^2^Lung Cancer Center, West China Hospital of Sichuan University, Chengdu, China; ^3^Department of Anesthesiology, West China Hospital of Sichuan University, Chengdu, China

**Keywords:** superior vena cava syndrome, thyroid cancer, superior vena cava reconstruction, tumor thrombus, vein to vein bypasses support, case report

## Abstract

**Background:** Thyroid cancer with massive invasion into the cervical and mediastinal great veins is extremely rare, and the surgical treatment is controversial, thus posing a great challenge for head and neck surgeons. Here, we report our successful experiences in reconstructing the superior vena cava (SVC) system to treat thyroid cancer with an extensive tumor thrombus growing intraluminally into the SVC.

**Case Presentation:** From September 2019 to September 2020, three patients with superior vena cava syndrome(SVCS) caused by tumor thrombus invasion from thyroid cancer were continuously included in this series. After preoperative evaluation, radical resection and reconstruction of the SVC system with expanded polytetrafluoroethylene (EPTFE) grafts were performed. In addition, bypass support from the right internal jugular vein to the right femoral vein was routinely prepared intraoperatively to prevent a rise in central venous pressure (CVP). Postoperatively, SVC-related syndrome improved immediately after the operation. Imaging examination showed good function of the reconstructed venous system. The patients recovered well with no surgical complications and remain under continuous follow-up.

**Conclusions:** Tumor growth into the SVC does not seem to be an absolute contraindication for surgery for thyroid carcinoma. Comprehensive treatment, including reconstruction of the SVC, is effective for relieving symptoms and preventing disease progression and is thus worth advocating. In addition, bypass support from the internal jugular vein to the femoral vein is easy to implement and can improve the safety of the operation.

## Background

Differentiated thyroid cancer (DTC) is the most common subtype of thyroid cancer, accounting for more than 95% of cases (1). Generally, most DTC patients have a very good prognosis with an overall 10-year relative survival rate of 90% (2). However, in some patients with advanced-stage DTC, extrathyroid extension and local invasion not only are associated with poor prognosis but also pose a great challenge for effective surgical treatment (3). Of these cases, superior vena cava syndrome (SVCS) caused by an extensive tumor thrombus growing intraluminally is extremely rare, and the therapeutic schedule and outcomes for such patients have been described as limited.

SVCS can present as headache, neck/face swelling, varicose veins, dyspnea, intracranial hypertension and some possibility of sudden death, such as that from pulmonary embolism (4). Traditional treatments, such as dieresis, radiotherapy and bypass grafts, may alleviate symptoms, but such treatments will not radically cure the underlying cause (5, 6). In recent decades, surgical reconstruction of the superior vena cava (SVC)in patients with extensive venous thrombosis has been widely adopted for cases not amenable to endovascular treatment, but the difficulty and high risk of surgical treatment still leads to ~4.5 to 14% mortality (7, 8). In this case series of three patients, we seek to describe our clinical experience of using expanded polytetrafluoroethylene (EPTFE) grafts for intraoperative reconstruction of the SVC system to treat thyroid cancer with an extensive tumor thrombus growing into the SVC; we also evaluate procedure-related outcomes.

## Case Presentation

In September 2019, we reported on our We Media the first case in our hospital of SVC reconstruction to treat SVCS due to thyroid tumor thrombus growing intraluminally. Then, two similar patients underwent successful SVC reconstruction. The detailed diagnosis and treatment of the patients were as follows. This study was approved by the Ethics Committee of West China Hospital of Sichuan University and was conducted in full accordance with ethical principles, including those stated in the World Medical Association Declaration of Helsinki (version 2002). Written informed consent was obtained from all participants for publication of this case report and any accompanying images.

### Surgical Procedures

**Figure 1:**
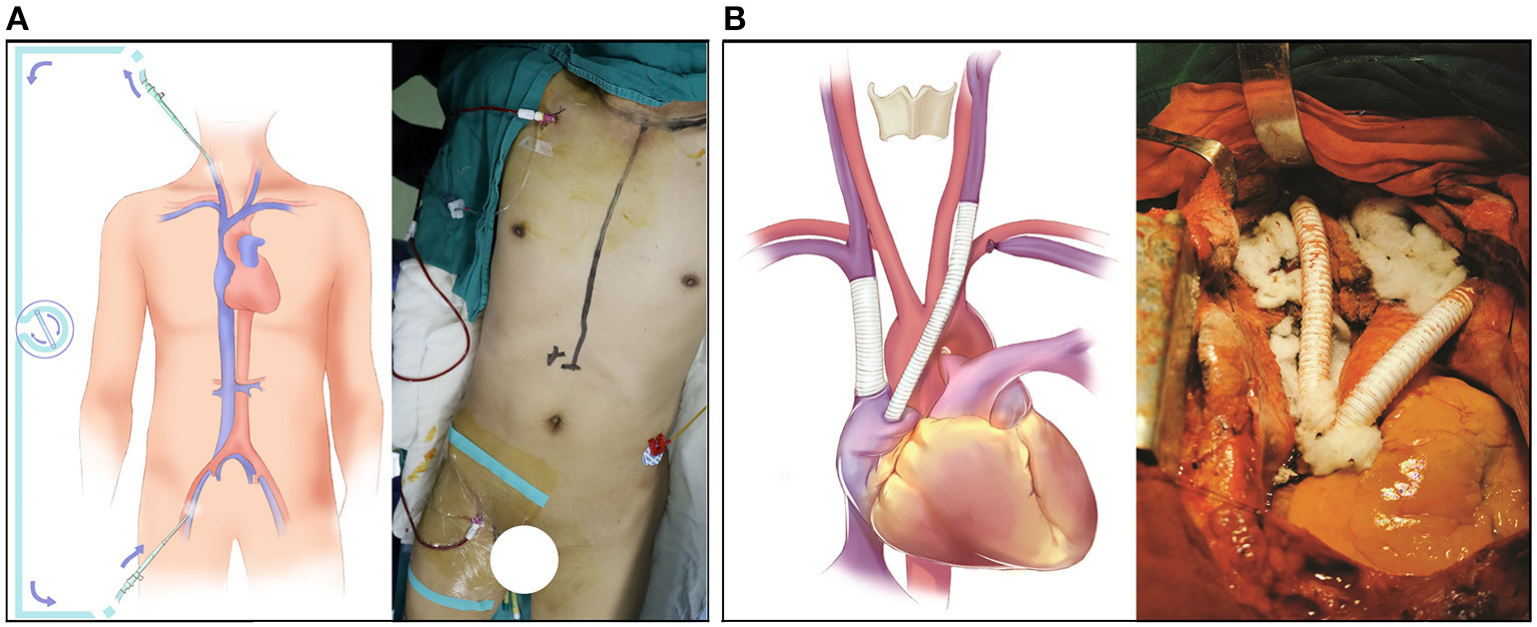
Surgical detail of the SVC resection and reconstruction. **(A)** The bypass support from the internal jugular vein to the femoral vein. **(B)** The reconstruction of the SVC system with an EPTFE graft.

After general anesthesia, both the right internal jugular vein and the right femoral vein were cannulated with 8.5 F arterial cannulas, and the two ends of the cannulas were connected by a one-way-valved catheter (Figure 1A). This process was performed under the guidance of bedside ultrasound to avoid the embolization site. Then, a 5F central venous catheter was inserted into the right internal jugular vein to monitor the internal jugular vein pressure continuously. Heparin 0.5 mg/kg was given before blocking the SVC, and this action was maintained for 200–250 s. After the SVC reconstruction was completed, the same amount of protamine and heparin were given. During the blocking period, if blood pressure decreased, m-hydroxylamine was intermittently used to maintain hemodynamic stability. Thus, the whole operative procedure was performed under veno-venous bypass support from the right internal jugular vein to the right femoral vein to reduce the pressure in the SVC system and prevent brain edema.

SVC reconstruction was performed via median sternotomy, and a bilateral transverse incision was performed above the clavicle to completely expose the SVC system. The whole operation went from left to right. First, the left brachiocephalic vein was blocked and cut off; then, the left brachiocephalic vein and right auricle were bridged with an EPTFE graft. During this process, it was important to minimize the compression or traction of the involved vessels to prevent the tumor thrombus from falling off. After cutting the left internal jugular vein, the intravascular tumor thrombus was cleaned, and the vessel was repeatedly washed with heparin saline to ensure that the blood flow was smooth and there was no foreign body in the vascular cavity. Then, the right atrial appendage was longitudinally clamped with a side-biting Satinsky clamp, and an incision (~15 mm) was made. The incision was dissected into the interior of the atrial appendage, and trabecular muscles were excised to improve inflow. The reconstruction between the left internal jugular vein and right auricle was performed with the EPTFE graft by using a parachute suture technique with 4-0 Prolene sutures. After the anastomosis was completed, the blockage of the distal end was released first to fill the artificial blood vessels with blood, and then the blockage of the proximal end was released to avoid the formation of an air embolism. With this process, regardless of the degree of SVC obstruction and the establishment of collateral circulation, we could block the main SVC calmly without worrying about the blocking time.

Then, the right brachiocephalic vein and SVC were blocked, and the intervening vessels that were invaded by the tumor thrombus were removed; then, the brachiocephalic vein and SVC were bridged with an EPTFE graft. Subsequently, the mediastinal tumor and the invaded SVC were completely freed and resected, respectively, and the whole reconstruction of the SVC was completed (Figure 1B).

### Patient 1

A 24-year-old man was admitted to our hospital for progressive enlargement of the anterior neck and dyspnea for 3 months. He had a history of a total thyroidectomy for papillary thyroid carcinoma (PTC) 1 year prior. Three months after the operation, the ultrasonography showed a tumor thrombus in the left subclavian vein and left internal jugular vein, so rivaroxaban 20 mg was given daily for anticoagulation. Subsequently, the anterior neck of the patient was gradually enlarged and was accompanied by a progressive aggravation of dyspnea. At the time of admission, the head, neck and upper trunk of the patient were seriously swollen.

**Figure 2:**
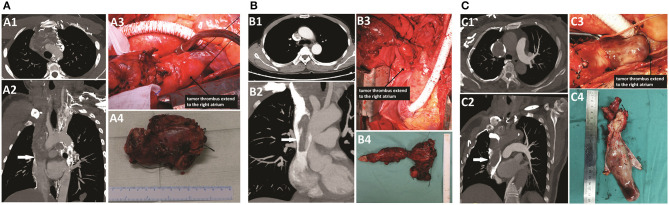
CTA scan and intraoperative findings of patients. **(A)** Patient 1: A1–A3, an extended tumor thrombus totally occupied the left brachiocephalic vein and the SVC, near the right atrium (white arrow). A4, the resected vessels invaded by the tumor thrombus. **(B)** Patient 2: B1–B3, an extended tumor thrombus occupied the left brachiocephalic vein and the SVC, near the right atrium, but the obstruction of the SVC segment was incomplete (white arrow). B4, the resected vessels invaded by the tumor thrombus. **(C)** Patient 3: C1–C3, an extended tumor thrombus totally occupied the right brachiocephalic vein and the SVC, near to the right atrium (white arrow). C4, the resected vessels invaded by the tumor thrombus.

Contrast-enhanced computed tomography (CT) showed a highly enhanced lesion measuring 5 cm in diameter on the anterior mediastinum; the lesion was accompanied by multiple 0.5–2 cm metastatic lung nodules (Supplementary Figure 1). Further investigation with CT angiography (CTA) showed filling defects in the proximal segment of the left internal jugular vein and left subclavian vein, left brachiocephalic vein and SVC only ~1 cm from the right atrium (Figure 2A). The serum thyroglobulin (Tg) was high (709.9 μg/L) with the normal level of thyroglobulin antibody (TgAb) under thyroid-stimulating hormone (TSH) suppression (0.431 mU/L). No other distant metastases were detected by whole-body bone scintigraphy and positron emission tomography CT (PET/CT), and no obvious abnormality was found by echocardiography (ECHO). Given the risk of brain edema with the development of SVCS and the risk of sudden death due to tumor embolism or obstruction of the tricuspid valve, surgical treatment with resection and reconstruction of involved vessels was chosen by the multidisciplinary team.

The intraoperative findings are shown in Figure 2A, and the surgical details and postoperative outcomes are listed in Table 1.

**Table 1:** Surgical details and postoperative outcomes of patients.

**Parameter**	**Patient 1**	**Patient 2**	**Patient 3**
Age (year)	24	51	68
Sex	M	M	F
Pathology	PDTC	FTC	FTC
Extension	IJV to RA	IJV to RA	IJV to RA
Operation time (h)	8	8	6.5
Blood loss (mL)	3000	4000	300
Diameter of EPTFE graft (mm)			
LIJV to RA	8	12	12
RIJV to SVC	14	14	12
Postoperative hospital stays (days)	17	18	20
Complications	NA	Hoarseness	NA
Outcome	Survived for 18 months (treatment with apatinib)	Survived for 12 months	Survived for 7 months

*M, male; F, female; PDTC, poorly differentiated thyroid carcinoma; FTC, follicular thyroid carcinoma; IJV, internal jugular vein; RA, right atrium; EPTFE, expanded polytetrafluoroethylene graft; LIJV, left internal jugular vein; RIJV, right internal jugular vein; SVC, superior vena cava; NA, not available*.

### Patient 2

The second patient was a 51-year-old man. He was admitted to our hospital for the presence of an enlarged anterior cervical mass for more than 4 years. He had a history of partial thyroidectomy in the local unit 5 years ago, but the detailed scope of resection and postoperative pathological results were unknown. Neck ultrasonography showed two gigantic masses measuring ~87 × 45 mm and 50 × 49 mm located in the left and right thyroid, respectively. The patient had no significant dysphonia, and laryngoscopy showed normal bilateral vocal cord activity. The results of fine-needle aspiration (FNA) in the left mass revealed some minute follicles, and consequently, a diagnosis of follicular thyroid carcinoma (FTC) was suspected. Further investigation with CTA (Figure 2B) showed that the left internal jugular vein was invaded by the left mass; filling defects were found in the left internal jugular vein, left brachiocephalic vein, and SVC and extended to the right atrium, while the obstruction of the SVC segment was incomplete. The right internal jugular vein and the upper segment of the brachiocephalic vein narrowed only due to compression of the mass. In addition, several nodules measuring 0.5–2 cm were detected in the lung. No other distant metastases were detected by whole-body bone scintigraphy and PET/CT. Residual thyroidectomy plus bilateral neck lymph node dissection and SVC reconstruction were planned.

During the operation, because the bilateral recurrent laryngeal nerve was too tightly wrapped by the tumor to be separated, we removed the bilateral recurrent laryngeal nerve at the same time. Then, because the neck was connected with the mediastinum by the incision, a tracheotomy was not performed to avoid sputum contamination of the mediastinum or even erosion of the EPTFE grafts. Therefore, bilateral posterior vocal cord resection was performed to ensure the postoperative respiratory function of the patient. No suspicious parathyroid glands were found in the operative area or in the excision tissues. The intraoperative findings are shown in Figure 2B.

### Patient 3

The third patient was a 68-year-old female. She was admitted to our hospital for the presence of an enlarged right anterior cervical mass for more than 1 year. She had no other discomfort but occasional dyspnea. Neck ultrasonography showed one gigantic mass measuring ~90 × 39 × 29 mm located in the right thyroid. Further investigation with CTA (Figure 2C) showed that the mass of the right thyroid lobe invaded the initial segment of the esophagus and compressed the trachea. Filling defects were found in the right internal jugular vein, right brachiocephalic vein, and SVC and extended to the right atrium. The results of FNA in the cervical mass and embolus of the right internal jugular vein revealed follicular cells with atypia of undetermined significance, and consequently, a diagnosis of FTC with invasion into the mediastinal great veins was suspected. The ECHO indicated an enlargement of the left atrium. A thyroidectomy, a right central and lateral neck dissection, and an SVC reconstruction were performed, and the intraoperative findings were shown in Figure 2C.

### Postoperative Results

The surgical details and postoperative outcomes of these three patients are listed in Table 1. The median operation time was 7.5 ± 0.9 h, with blood loss being ~3,000 mL in patient 1, 4,000 mL in patient 2, and 300 mL in patient 3, while the corresponding intraoperative blood transfusion was also given. The diameter of the EPTFE graft was 8–12 mm between the left internal jugular vein and right atrium and 12–14 mm between the right internal jugular vein and proximal SVC.

The SVC-related syndrome improved immediately in all three patients after the operation, and there were no intraoperative or postoperative complications except for patient 2. This patient underwent bilateral posterior vocal cord resection after the bilateral recurrent laryngeal nerve was removed. Hence, he presented with hoarseness but no obvious dyspnea. All patients underwent CT scan and ECHO to confirm that the reconstructed venous system was functioning well and to exclude potential abnormalities, such as excessive pleural and pericardial effusion. These three patients recovered uneventfully, the lengths of postoperative hospital stays ranged from 17 to 20 days, and the median time from operation to discharge was 18.3 ± 1.5 days.

### Patient Survival at Follow-Up

The postoperative pathological results of patients 1, 2, and 3 showed poorly differentiated thyroid cancer (PDTC), FTC and FTC, respectively. So far, all patients have been closely followed up in our outpatient department, and no patient has died. Length of follow-up ranged from 7 to 18 months after surgery. In addition, because the postoperative pathology in patient 1 showed PDTC with wild-type BRAF and TERT and there were several metastases in the lung, he continued to receive targeted therapy with apatinib 1 month after surgery. At that time, the laboratory examination revealed Tg 408.0 μg/L, which was significantly lower than the Tg level before the operation. Five months later, the serum Tg level of the patients was further reduced (173.8 μg/L). Although the diameter of pulmonary metastasis did not change significantly, there were clear cavities in the focus (Supplementary Figure 1), thus indicating that the focus responded well to apatinib (9). The patient had no obvious adverse events caused by apatinib, and liver function and coagulation function were normal and are still in continuous follow-up.

## Discussion

SVCS caused by extended tumor thrombus is extremely rare in patients with thyroid cancer. Two reviews reported that 54 patients had major vessel invasion by thyroid cancer from 1879 to 2018 (10, 11). Of these patients, 28 cases present as SVCS, and only six of the patients involved received aggressive resection and reconstruction treatment; however, only three patients survived for a long time (12–16). The details of published reports regarding the reconstruction of the SVC to treat SVCS due to tumor thrombi from thyroid cancer are listed in Table 2. The high mortality of the surgery highlights the importance of surgical techniques. However, the operation has not been described in detail in previous studies, and how to standardize the operation and what surgical techniques need to be considered are still unclear. In this report, we described three cases of successful reconstruction of the SVC to treat SVCS due to tumor thrombi from thyroid cancer, and we hope that our surgical details and initial outcomes can serve as a reference for treating similar patients.

**Table 2:** Review of published reports regarding SVC reconstruction to treat thyroid cancer with tumor thrombi.

**References**	**Country**	**Patients**	**Pathology**	**Extension**	**Method for maintaining CVP**	**Reconstruction materials**	**Outcome**
Hasegawa et al. (13)	Japan	78 F	PTC	IJV to RA	CPB	EPTFE graft	Died 36 days postoperatively with respiratory failure
Motohashi et al. (14)	Japan	64 F	PTC	IJV to SVC	Not mentioned	EPTFE graft	Survived for 24 months
Wada et al. (16)	Japan	74 F	PTC	IJV to SVC	Temporary bypass between the left brachiocephalic vein and the right auricle	Autologous tissue (resected left brachiocephalic vein)	Died 19 months postoperatively with lung metastasis with pleural effusion
		64 M	FTC	IJV to SVC	Not mentioned	Autologous pericardial patch	Survived for 8 months
Sugimoto et al. (15)	Japan	61 M	PTC	IJV to RA	CPB	EPTFE graft	Died 12 days postoperatively with renal failure
Niederle et al. (12)	Austria	53 F	FTC	IJV to RA	Not mentioned	EPTFE graft	Died 8 months postoperatively with occlusion of the graft
Chen W, et al. (present)	China	24 M	PDTC	IJV to RA	Bypass from the internal jugular vein to the femoral vein	EPTFE graft	Survived for 18 months
		51 M	FTC	IJV to RA	Bypass from the internal jugular vein to the femoral vein	EPTFE graft	Survived for 12 months
		68 F	FTC	IJV to RA	Bypass from the internal jugular vein to the femoral vein	EPTFE graft	Survived for 7 months

*M, male; F, female; PDTC, poorly differentiated thyroid carcinoma; FTC, follicular thyroid carcinoma; IJV, internal jugular vein; RA, right atrium; EPTFE, expanded polytetrafluoroethylene graft; LIJV, left internal jugular vein; RIJV, right internal jugular vein; SVC, superior vena cava; CVP, central venous pressure; NA, not available*.

The surgical indication and long-term mortality for SVCS reconstruction to treat malignant disease are controversial. Picquet's study (8) reported that SVC reconstruction can significantly improve the symptoms and quality of life for patients with thoracic malignant tumors, and the long-term mortality was due mainly to the extension and severity of the cancer but not related to SVC reconstruction. Similar results were also reported in Shargall's study (17). However, in Spaggiari's study, pneumonectomy and complete SVC resection with prosthetic replacement were reported as independent risk factors for increased risk of death, while this result was probably limited by surgical experience and by the anesthesiological and technical devices of the era (7). In sum and in consideration of the experience at our center, we suggest that surgical reconstruction can be considered according to the following criteria: (1) the neoplasm can be completely resected, and no distant metastatic lesions are detected; and (2) the patient is in good physical condition and can tolerate thoracotomy. However, to delay the progress of the disease and also relieve the acute or severe symptoms, SVC reconstruction could also be considered even if these patients do not meet the above indications (18). Finally, the surgical risk and benefit should be fully and rationally considered because SVC reconstruction requires skilled surgical experience and special anesthesia and technical support.

Avoiding brain edema after SVC occlusion is one of the important facets of a successful operation. Previous studies have detected that SVC clamping for <45 min is well-tolerated by the brain (19), while patients with chronic SVCS can tolerate the obstruction longer because of the sufficient collateral circulation (17, 20). Measures, such as controlled lowering of blood pressure and ice cap cooling before SVC occlusion, have been suggested in some previous studies, but the controllability of these measures is poor, and the effect is not obvious. Cardiopulmonary bypass (CPB) support is a classical method to relieve SVC obstruction (21, 22), but this method is expensive and requires systemic heparinization, which may lead to several potential complications, such as empyema, bleeding and coagulopathy (23). Moreover, CPB is performed mainly in the operating field and may thus affect the surgical process and increase the difficulty of vascular anastomosis; in addition, establishing an artificial channel also increases the trauma and complexity of the operation. In this study, according to the pulse pressure difference between the SVC and femoral vein, we describe a method of internal-jugular-vein–to–femoral-vein-bypass support, which is easy to operate, pollution-free, and controllable and does not need systemic heparinization. This method can effectively relieve the SVC pressure and maintain stable hemodynamics during the operation. To the best of our knowledge, the first reported use of this method was at our center for SVC resection and reconstruction in patients with lung cancer (24), and this method has been reported to be used for SVC reconstruction in patients with other tumors (25). Of course, the disadvantages of this method cannot be ignored due to the restriction of the bypass channel. When the SVC pressure rises sharply, it cannot drop rapidly; however, after a certain time, this pressure will decrease to the ideal range, and there is no obvious influence on the operation.

The other key point is the selection of graft length and diameter. If the graft is too long, it is easy to twist and can be easily squeezed by the closed sternum; if the graft is too short, the tension of the anastomosis is large, thus easily causing pinhole hemorrhage, and the graft near the anastomotic opening may be pulled and deformed and may thus be followed by stenosis and thrombosis. Therefore, the end-to-end distance should be measured before anastomosis to estimate the graft length. In addition, whether the diameter of the graft is too small or too large, it is easy to cause the steal phenomenon because of the hemodynamic abnormality, thus leading to reduced blood flow in the thinner graft followed by thromboembolism. Hence, according to the original vein diameter, an 8–12 mm diameter in the left brachiocephalic vein and a 12–14 mm diameter in the SVC of grafts were suggested (26).

Some studies recommend the use of autologous vein grafts, such as the superficial femoral vein (27), great saphenous vein (28) and pericardium (16), but we prefer the EPTFE graft. Because the diameters of the great saphenous vein and superficial femoral vein are much smaller than that of the SVC, too narrow of a graft can easily cause hemodynamic changes and decrease the blood flow velocity, thus increasing the risk of thrombosis. The catheter composed of pericardial suture also has the risk of thrombosis due to the long suture. Moreover, because the pericardium is thin and the venous pressure is not high, with the proliferation and compression of the mediastinal scar tissue, the catheter can easily collapse and cause stenosis. Instead, because the inner wall of EPTFE is smooth, this material can ensure the patency of the vascular cavity and will not easily form thrombi. In addition, the “ring” structure has a certain degree of anticompression ability, which can avoid the expansion or collapse of the vascular cavity. Therefore, EPTFE has become the preferred choice for many clinicians (8, 26, 29).

## Conclusions

In conclusion, we report three successful SVC reconstructions in patients with the rare case of SVCS caused by thyroid cancer. Even if the tumor grows into the SVC, this growth does not seem to be an absolute contraindication for surgery for thyroid carcinoma. Comprehensive treatment, including reconstruction of the SVC, is effective for relieving symptoms and preventing the development of disease and is thus worth advocating. In addition, bypass support from the internal jugular vein to the femoral vein is easy to implement and can improve the safety of the operation.

## Data Availability Statement

The raw data supporting the conclusions of this article will be made available by the authors, without undue reservation.

## Ethics Statement

The studies involving human participants were reviewed and approved by the Ethics Committee of West China Hospital of Sichuan University. The patients/participants provided their written informed consent to participate in this study.

## Author Contributions

WC analyzed the data and wrote the first draft. JL, YW, XT, BL, ZL, and QZ performed the surgeries as a multidisciplinary team. ZL and QZ revised the article critically for important intellectual content. ZL is the guarantor and is directly responsible for the manuscript. All authors substantially contributed to the conception and design of this article and gave final approval for the version for publication and agree to be accountable for all aspects of the work.

## Conflict of Interest

The authors declare that the research was conducted in the absence of any commercial or financial relationships that could be construed as a potential conflict of interest.

## Abbreviations

DTC: differentiated thyroid cancer
SVCS: superior vena cava syndrome
SVC: superior vena cava
EPTFE: expanded polytetrafluoroethylene
PTC: papillary thyroid carcinoma
RAI: radioiodine ablation
CT: computed tomography
CTA: computed tomographyangiography
Tg: thyroglobulin
TgAb: thyroglobulin antibody
PET/CT: positron emission tomography/computed tomography
ECHO: echocardiography
FNA: fine-needle aspiration
FTC: follicular thyroid carcinoma
PDTC: poorly differentiated thyroid cancer
CPB: cardiopulmonary bypass.
