# Supplementary figures and images for: Case Report: Superior Vena Cava Resection and Reconstruction for Invasive Thyroid Cancer: Report of Three Cases and Literature Review
Source: Front Surg. 2021 Jun 1;8:644605. doi: 10.3389/fsurg.2021.644605 (PMC8204692; doi:10.3389/fsurg.2021.644605)

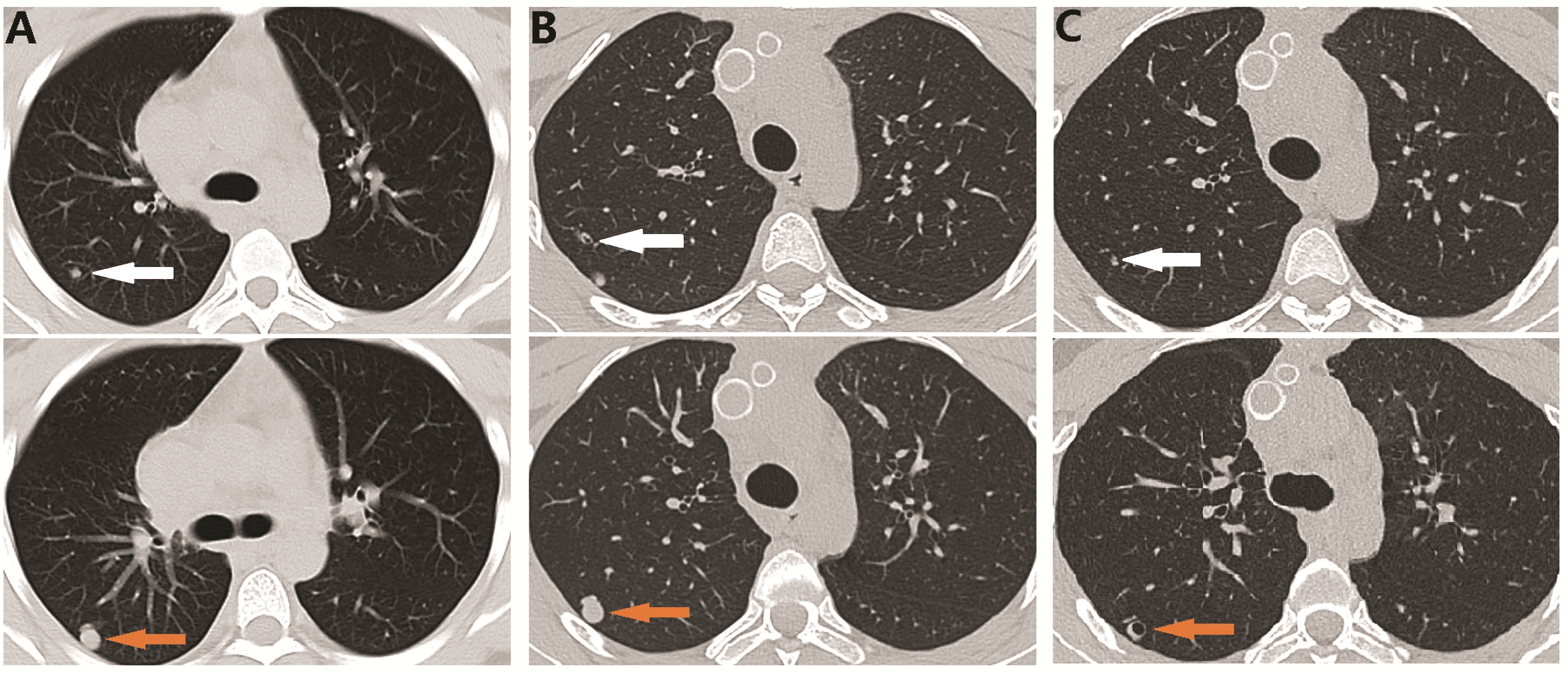

Supplement: Supplementary file 1 [file Image_1.TIF]
