# Supplementary material for: Case Report: Superior Vena Cava Resection and Reconstruction for Invasive Thyroid Cancer: Report of Three Cases and Literature Review
Source: Front Surg. 2021 Jun 1;8:644605. doi: 10.3389/fsurg.2021.644605 (PMC8204692; doi:10.3389/fsurg.2021.644605)
Supplement: Supplementary file 2 [file Table_1.DOCX]

Supplementary Material

**Supplementary** **figure legends：**

**Supplementary Figure 1.** Chest CT scans of patient 1. A: Two nodules were noted in the right lower lobe. B: One month after apatinib therapy, the nodule in the upper part (white arrow) developed cavitation, but there was no significant change in the dominant nodule (yellow arrow). C: Four months after apatinib therapy, the diameter of the upper nodule was significantly reduced, and a cavity developed in the dominant nodule.
